# Substance use disorder, the workforce, and treatment quality for minoritized populations: a systematic review

**DOI:** 10.1186/s13011-025-00656-8

**Published:** 2025-06-19

**Authors:** Hannah L. Maxey, Brittany J. Daulton, Rebekka Boustani, Kelsey E. Binion

**Affiliations:** 1 Department of Family Medicine, Indiana University School of Medicine, Lockefield Village, 980 Indiana Ave, Indianapolis, IN 46202 USA; 2 Department of Health Policy and Management, Indiana University Richard M. Fairbanks School of Public Health, 1050 Wishard Blvd, Indianapolis, IN 46202 USA

**Keywords:** Substance use disorder, Workforce, Health policy, Health disparities

## Abstract

Substance use disorder remains a United States public health concern, particularly for minoritized populations. This systematic review explores the relationship between workforce diversity, cultural competency, and the quality of substance use disorder treatment for minoritized populations. Studies published between 2003 and 2023 were analyzed and nine studies met inclusion criteria. Findings reveal conflicting evidence about the impact of workforce diversity and cultural competency on quality outcomes. While some studies suggest positive associations, others report no relationship or adverse effects. Moreover, inconsistencies in research methodology limited comparative analyses. The literature predominantly focuses on Hispanic/Latinx populations, leaving gaps in understanding other minoritized populations’ experiences. Despite national initiatives promoting health equity and diversity, federal research funding regarding substance use disorder treatment for minoritized populations remains limited. This review calls for further research to inform policy and practice, emphasizing multi-agency collaboration, standardized measures of cultural competency, and targeted funding to address disparities in treatment quality outcomes for minoritized populations.

**Supplementary Information:**

The online version contains supplementary material available at 10.1186/s13011-025-00656-8.

## Introduction

Substance use disorder (SUD) is a leading cause of death and disability among Americans, disproportionately affecting minoritized populations. [1] SUD is a complex, treatable condition but successful treatment depends on a workforce capable of delivering high-quality care [2–4].

Building and sustaining a healthcare system that delivers quality care consistently and equitably has been a long-standing focus in the United States (U.S.) [5–7]. Recommendations to achieve health system goals have included targeted health workforce initiatives. Although progress has been made in developing strategies for improving healthcare, including SUD, quality of care are inconsistent, especially among racially minoritized populations [8–10]. With the consequences of SUD at crisis levels, policies aimed at developing equitable healthcare outcomes are imperative [1].

### Healthcare Quality and the Workforce

The healthcare workforce’s role in quality of care has long been acknowledged because of its impact on the U.S. population’s health outcomes and resources. In 2001, the Institute of Medicine (IOM) wrote *Crossing the Quality Chasm*, a seminal report focused on reducing medical errors and containing costs to enhance healthcare quality [5]. Workforce recommendations focused on modernizing health profession education and practice to support higher quality and better-coordinated care, containing costs and reducing errors (Table 1).

**Table 1:** Foundational reports and relevant recommendations

Report Title	Year	Summary	Relevant Report Recommendation
**Crossing the Quality Chasm: A New Health System for the 21 st Century**	**2001**	A call for a sweeping redesign of the American health care system to close the quality gap	**Recommendation 12 (p. 208)**
			Modernize health profession education: • Leverage technology for care delivery • Measure quality, including processes and outcomes • Promote interprofessional collaboration • Understand the effect of social determinants of health
**Unequal Treatment: Confronting Racial and Ethnic Disparities in Health Care**	**2003**	An examination of healthcare disparities and proposed interventions to integrate cultural sensitivity within the health professions	**Recommendation 5–3 (p. 186)**
			Integrate cross cultural education into the training of health professionals to improve cultural competence
			**Recommendation 6–1 (p. 214)**
			Increase the proportion of underrepresented minorities to diversify the health workforce
**Improving the Quality of Health Care for Mental and Substance-Use Conditions**	**2006**	A multifaceted and comprehensive strategy for improving health care for mental and substance-use conditions	**Recommendation 7–1 (p. 317–318)**
			Congress should establish a Council of Mental and Substance-Use Care Workforce: • Identify specific clinical competencies • Fund programs that research workforce issues like diversity and cultural relevance • Providing continuing assessment of workforce trends

In 2006, *Improving the Quality of Health Care for Mental and Substance-Use Conditions* was published as a sector-specific follow-up report [6]. Workforce recommendations called for the U.S. Congress to establish a Council on the Mental Health and Substance Use Workforce to oversee national initiatives to strengthen the workforce, including formalizing clinical competencies and research funding (Table 1) [6].

### Healthcare disparities and the workforce

Health disparities also emerged as a top priority in the early 2000’s. In 1999, the U.S. Surgeon General issued a report highlighting the racial and ethnic disparities in mental health and SUD [11]. *Unequal Treatment,* the landmark report on racial and ethnic disparities across the U.S. healthcare system, was published in 2002 [7]. Both reports provided evidence of racial and ethnic differences in quality, described contributing factors, and delivered workforce recommendations to increase diversity and improve cultural competence (Table 1) [7].

### Workforce diversity and racial/ethnic concordance

Recommendations to improve workforce diversity seek to increase racial/ethnic concordance between clinicians and patients thereby improving likelihood of shared cultural beliefs, values, and experiences, enabling effective communication, fostering trust, and improving quality [7, 12]. Research indicates racial concordance is associated with increased patient satisfaction, but findings are inconclusive for more direct measures of quality, such as treatment outcomes [12–19].

### Cultural competence

Although it would be ideal for patients to select a racially/ethnically concordant provider, this does not ensure shared cultural beliefs, values, or a culturally competent workforce. Cultural competency in healthcare has been defined varyingly. Generally, cultural competence involves knowledge of and respect for values, attitudes, and beliefs across different cultures and integrating that knowledge into culturally responsive healthcare services [20, 21].

#### Present Study

Health policies aim to equitably improve health and well-being, and progress has been made since the publication of *Crossing the Quality Chasm*. Workforce-related policy recommendations (Table 1) have been adopted by the healthcare system. Telehealth is a ubiquitous modality of care delivery [22]; nationally accepted quality measures are used to monitor health systems and inform policy [23]; interprofessional education is a priority [18]; team-based care is commonplace [24]; and social determinants of health are integrated into patient care [25]. Yet, health disparities persist in the United States and must be addressed.

The problem is that despite numerous efforts and calls to action, the role of workforce factors such as cultural competency, diversity, and concordance on the quality of SUD treatment for minoritized populations remains underexplored, contributing to ongoing disparities in treatment outcomes. The unprecedented increase in SUD-related deaths, characterized by significant racial disparities, calls for a national commitment to delivering quality treatment for minoritized populations [1, 26]. Researchers and policymakers generally agree on increasing workforce diversity and cultural competency to reduce health disparities and improve treatment quality for minoritized populations. Although multiple U.S. Presidents and their administrations, congressional committees, researchers, philanthropic organizations, and advocates have championed efforts to address the healthcare workforce [27–32], the role of these workforce factors on SUD treatment quality for minoritized populations has not properly been explored. Therefore, the purpose of the current study was to explore the existing literature examining the relationship between workforce factors (i.e. cultural competency, diversity, and concordance) and the quality of SUD treatment for minoritized populations to develop updated calls to action based on the state of the research today.

#### Study Data and Methods

**Fig. 1:**
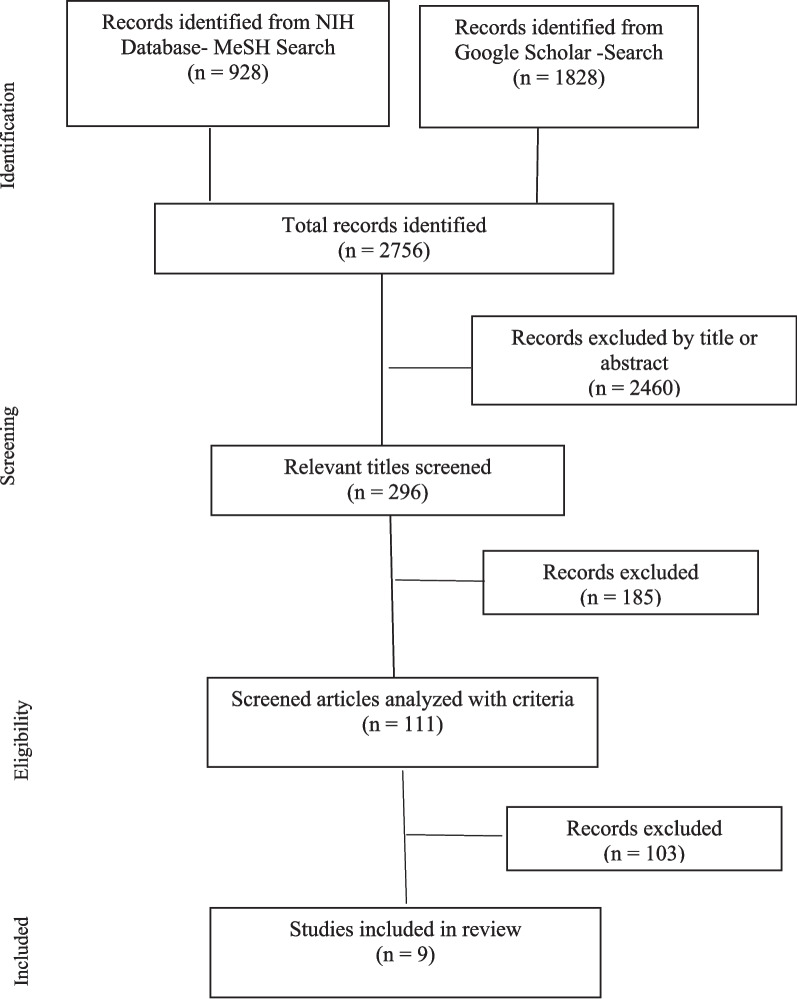
PRISMA flow chart

The study followed the Preferred Reporting Items for Systematic Reviews and Meta-Analyses (PRISMA) checklist (Fig. 1) [33].

### Identification and Selection of Studies

Multiple queries of MeSH (Medical Subject Headings) terms (appendix Table A1) were performed using the National Library of Medicine (NLM) and Google Scholar to identify peer reviewed studies available online, published between 2003 and 2023, using human subjects, and the English Language. Publications with relevant titles and abstracts were identified for further review.

Seminal and foundational reports have long reported recommendations for the health care workforce’s role in quality of care, including increasing workforce diversity and cultural competence in order to decrease disparities and inequities [5, 7]. Therefore, this study focuses on diversity characteristics of the workforce in providing SUD treatment for minoritized populations, specifically diversity in staff, cultural competence, and/or concordance (i.e. matching between patient and provider based on an identified characteristic).

The following selection criteria were used to identify articles for synthesis: 1) minoritized populations receiving SUD treatment; 2) workforce characteristics (diversity, cultural competence, or both); and 3) SUD treatment quality as measured by patient reported outcomes and experience (adherence, time to treatment, self-reported substance use); (Table 2). Previous research indicates patients’ perception of care (i.e. satisfaction, adherence, etc.) can be used as an indirect measure of quality potentially associated with direct measures of treatment outcomes [7, 12–16]. Patients satisfied with their care are typically more likely to adhere to or complete treatment programs [34]. The current study focuses on perceptions of care through patient experience variables, categorized as adherence to treatment (i.e. completion and retention), treatment logistics (i.e. wait time to receive treatment), and frequency of substance use.

**Table 2:** Literature included in systematic literature review

Author, year	Title	Minoritized Population of Interest (racially or ethnically) and Sample Size (n)	SUD Treatment Outcome (biological marker, program, patient, clinician, reported)	Outcome Data Source	SUD Treatment Workforce (Diversity or Cultural Competence)	SUD Treatment Workforce Data Source	Overall Findings
Guerrero et al., 2022 [28]	Workforce Diversity and Disparities in Wait Time and Retention Among Opioid Treatment Programs	African American and Latino (Both) n = 162 in 2000; 300 in 2017	Wait time to treatment & treatment retention at 3 months (program reported)	National Drug Abuse Treatment Service Survey	Representation of African American and Latino Staff within SUD Provider Organizations (Both)	National Drug Abuse Treatment Service Survey	Greater workforce diversity associated with higher wait times (Black) and lower SUD treatment retention (Latino)
Guerrero et al., 2017 [29]	Does the Implementation of Evidence-Based and Culturally Competent Practices Reduce Disparities in Addiction Treatment Outcomes?	Latino (Ethnically) n = 153 providers; 15,412 clients	Treatment completion (patient reported, clinician reported)	Multiple datasets described in Guerrero, Andrews, Harris, et al. (2015)	Organizational cultural competency using a tool which includes representation of racial and ethnic minorities among staff as an indicator (Both)	Manager reported cultural competence using the Cultural Competence Self-Assessment Questionnaire (CLAS)	Greater cultural competence, including workforce diversity, was not associated with completion of treatment
Ruglass et al., 2014 [30]	Racial/Ethnic Match and Treatment Outcomes for Women with PTSD and Substance Use Disorders Receiving Community-Based Treatment	Black people (Racially) n = 224 clients	30-day frequency of use (patient reported)	Addiction Severity Index (ASI-Lite)	Racial matching to patient/study participant (Workforce Diversity)	Self-reported demographic information	Matched participants reporting zero use at baseline were more likely to report high use post treatment than unmatched participants. Matching had no effect on heavy users
Guerrero, 2013 [31]	Enhancing Access and Retention in Substance Abuse Treatment: The Role of Medicaid Payment Acceptance and Cultural Competence	African American and Latino (Both) n = 13,328 clients	Wait time to treatment and treatment retention (patient reported; clinician reported	Multiple datasets described in Guerrero, Andrews, Harris, et al. (2015)	Organizational cultural competency using a tool which includes representation of racial and ethnic minorities among staff as an indicator (Both)	Manager reported cultural competence using the Cultural Competence Self-Assessment Questionnaire (CLAS)	Diversity of staff, as an organizational indicator of cultural competence, was not associated with outcomes. Staff personal involvement in the community was associated with reduced wait times and increased retention
Guerrero et al., 2012 [32]	Do Cultural and Linguistic Competence Matter in Latinos’ Completion of Mandated Substance Abuse Treatment?	Latino (Ethnically) n = 63 providers; 5,150 cients	Treatment completion (clinician reported)	CalOMS database	Organizational cultural competence, including workforce diversity (Both)	Common-program level measures (National Drug Abuse Treatment Service Survey; National Standards for Culturally and Linguistically Appropriate Services (CLAS)	Cultural competence, including workforce diversity was not associated with treatment completion. Availability of translators was associated with treatment completion
Guerrero & Andrews, 2011 [33]	Cultural Competence in Outpatient Substance Abuse Treatment: Measurement and Relationship to Wait Times and Retention	African American and Latino (Both) n = 618 treatment programs	Wait time to treatment and treatment retention (program reported)	National Drug Abuse Treatment Service Survey (1995)	Organizational cultural competence, including workforce diversity and managers culturally sensitive beliefs (Both)	Common-program level measures (National Drug Abuse Treatment Service Survey; National Standards for Culturally and Linguistically Appropriate Services (CLAS)	Found some association between managers culturally sensitive beliefs and outcomes, but not the overall cultural competence practices, including workforce of the program
Suarez-Morales et al., 2010 [34]	Do Therapist Cultural Characteristics Influence the Outcome of Substance Abuse Treatment for Spanish-Speaking Adults?	Latino (Ethnically) *n* = 16 providers; 235 clients	Treatment participation and frequency of use (patient reported)	Treatment utilization form	Ethnic concordance with patient/participant (Workforce Diversity)	BiCultural Involvement Questionnaire (BiQ)	Ethnic matching was not associated with treatment outcomes
Field & Caetano, 2010 [35]	The Role of Ethnic Matching Between Patient and Provider on the Effectiveness of Brief Alcohol Interventions with Hispanics	Latino (Ethnically) n = 537 clients	Frequency of use (patient reported)	Program survey and telephone interviews	Ethnic concordance with patient/participant (Workforce Diversity)	Self-reported demographic information	Ethnic matching associated with improved outcomes
Flicker et al., 2008 [36]	Ethnic Matching and Treatment Outcome with Hispanic and Anglo Substance-Abusing Adolescents in Family Therapy	Latino (Ethnically) n = 9 providers; 86 participants	90-day frequency of use, urine screen (Patient reported, Biological Marker)	Timeline follow-back interview; collection of specimen	Ethnic concordance with patient/participant (Workforce Diversity)	Self-reported demographic information	Matching associated with reduction in frequency of use

### Identification of Funding Source

To explore the funding landscape of workforce diversity and SUD treatment, funding sources were identified. The Research Portfolio Online Reporting Tool (RePORTER) was used with identification numbers for National Institute of Health (NIH) awards. When grant numbers were unavailable, RePORTER was used to locate the grant using the PubMed Central ID number.

### Limitations

Searches conducted and filters used could be limiting. Therefore, not every relevant study may have been identified. The search potentially excludes relevant articles if the terms are not found in the title or abstract. Additionally, due to the heterogeneity of the research meta-analysis was not utilized. A meta-analysis would be inappropriate because the data do not meet statistical assumptions. Finally, several inconsistencies have been reported in the literature. Researchers used various measures and definitions in their research, which created difficulty comparing results across studies.

Other limitations to the current study are related to its specific focus on the workforce providing SUD treatment. Literature on the broader behavioral health workforce, specific SUD services and approaches, and settings of care were not part of the review criteria. Therefore, the findings of this review can only be applied to the SUD workforce.

## Results

The initial search included 64 combinations of search terms, yielding 2,756 hits. This included 296 relevant titles (Fig. 1). Nine publications were selected for synthesis, across which variations in the populations studied (both size and demographics), workforce diversity characteristics, and quality outcomes measures existed.

Study designs utilized across the included literature also varied. Most of the research synthesized was from a secondary analysis, utilizing retrospective data from public data monitoring, clinical records, or large standardized data sets, as opposed to intervention studies of comparative analyses [35–43]. Five studies examined ethnically minoritized populations, while one examined racially minoritized populations, and three examined both [35–43]. The three studies that examined both included Latino and black participants [35, 39, 44], the ethnically minoritized populations were all Latino participants, [36, 38, 41–43] and the racially minoritized populations were exclusively black participants. [40] No other minoritized populations were included in the reviewed studies. Additionally, the studies included various sample size numbers (n) of both clients and providers with a range from 86 to more than 15,000. Most of the literature included were secondary analyses of large data sets, creating large sample size availability.

With various study designs, various treatment quality outcomes were also reported, such as frequency of substance use, treatment logistics, and adherence with varying data sources and definitions. Two articles focused on adherence as the single indicator of quality, [38, 41] two articles focused on substance use as a single indicator, [42, 43] while two measured both adherence and use, [36, 40] and three measured adherence and logistics of treatment. Most outcomes were measured using patient self-report or clinician, but one source did include a biological marker (urine screening) to validate self-reported use [43].

Workforce diversity was also explored, as well as cultural competence or racial concordance, in relation to treatment quality for minoritized populations. Five articles studied both workforce diversity and cultural competence [35–39] and four examined only workforce diversity [40–43]. While three studies a measured racial/ethnic concordance or matching in SUD treatment [18, 36, 43, 45]. Other workforce characteristics were explored such as, such as managerial culturally sensitive beliefs, language competency, and connection to community resources (Table 2) [35, 37, 41, 42].

Diversity and cultural competence were operationalized in various ways throughout the included literature. Most often, workforce cultural competence was measured by manager or organizational self-reported cultural competence, typically utilizing the CLAS Assessment Questionnaire [35–38]. Otherwise, included articles utilized demographic information through other survey instruments, such as the BiCultural Involvement Questionnaire (BiQ), [41] percentage of staff that were black and/or Latino, [39] or self-reported demographic information [40, 42, 43]. Notable variations were also observed across the findings. Four studies examined ethnic or racial matching and treatment outcomes [40–43]. Matched participants showed some associations with frequency of use [42, 43]. But, there was no association between matching and other treatment quality outcomes (participation, retention, wait time) [40, 41]. In one instance, racially matched participants presented increased substance use after treatment [40]. Studies exploring workforce, organizational, or staff diversity found no or negative association with treatment outcomes [35–39]. However, studies exploring other workforce factors found availability of translators and linguistic competence to be associated with increased treatment completion for first-time Latino clients [36]. Providers’ community involvement was also associated with reduced wait times for treatment and increased retention [37].

Seven of the nine articles cited NIH funding, while one article cited funding from a non-federal source [37]. Only one grant specified workforce diversity and SUD treatment as primary objectives (Table 3) [40].

**Table 3:** Funding sources of included studies

Author, year	Funding Source 1	Study Purpose 1	Funding Source 2	Study Purpose 2	Funding Source 3	Study Purpose 3
Guerrero et al., 2022 [39]	R01 MD014639/MD/NIMHD NIH HHS	Determine the role of culturally competent strategies, and in particular workforce diversity, to improve treatment access				
Guerrero et al., 2017 [38]	R01 DA038608/DA/NIDA NIH HHS	Examine the extent to which increases in acceptance of public health insurance (ACA), leads to increases in quality of care in community-based SUD treatment	R25 MH080916/MH/NIMH NIH HHS	Five years of support to build, maintain, and sustain an innovative mentoring network for implementation science in mental health		
Guerrero et al., 2013 [37]	Larson Fund for Innovative Research & Teaching (Hamovitch Center for Science in the Human Services)	Unable to locate more information				
Guerrero et al., 2012 [36]	No additional information available					
Guerrero & Andrews, 2011 [35]	F31 DA024564/DA/NIDA NIH HHS	Examine organizational conditions that contribute to the implementation of cultural competence; and examine the process and dynamics that accompany different implementation levels and ethnic minority clients'treatment outcomes	3U10DA013038-10S2/DA/NIDA NIH HHS	To maintain and expand a highly successful infrastructure for drug abuse treatment research		
Ruglass, et al.,2014 [40]	K24 DA022412/DA/NIDA NIH HHS	Develop and test new treatment interventions that have the potential to significantly improve the outcome of drug use disorders, advancing them across the spectrum from efficacy to effectiveness	U10 DA013035/DA/NIDA NIH HHS	Evaluate whether the efficacy of the newer therapies generalizes to"real world"treatment settings and to disseminate these treatments and foster their appropriate use		
Suarez-Morales et al., 2010 [41]	U10 DA013720-10S3/DA	Develop and implement protocols that test the effectiveness of treatments across a broad range of community-based treatment settings with diverse patient populations, and to disseminate knowledge in a manner that improves the practice of drug abuse treatment	L60 MD000403/MD/NIMHD NIH HHS	Unable to locate more information		
Field & Caetano, 2010 [42]	R01 013824/PHS HHS/United States	Evaluate the efficacy of brief alcohol interventions in the emergency care setting among various ethnic groups	R01DA013350/DA/NIDA NIH HHS	Evaluate treatment efficacy for adolescent substance use disorders and HIV risk behavior among Anglo and Hispanic subgroups		
Flicker et al., 2008 [43]	R01AA12183/AA/NIAAA NIH HHS	Use a randomized clinical trial for adolescent problem drinking to examine treatment outcomes for four intervention approaches			R01DA009422/DA/NIDA NIH HHS	Clinical trial examining effectiveness of intervention approaches. Will also examine client and therapist characteristics and treatment operations which may influence treatment outcome
Field & Caetano, 2010 [42]	R01 013824/PHS HHS/United States	Evaluate the efficacy of brief alcohol interventions in the emergency care setting among various ethnic groups	R01DA013350/DA/NIDA NIH HHS	Evaluate treatment efficacy for adolescent substance use disorders and HIV risk behavior among Anglo and Hispanic subgroups		
Flicker et al., 2008 [43]	R01AA12183/AA/NIAAA NIH HHS	Use a randomized clinical trial for adolescent problem drinking to examine treatment outcomes for four intervention approaches			R01DA009422/DA/NIDA NIH HHS	Clinical trial examining effectiveness of intervention approaches. Will also examine client and therapist characteristics and treatment operations which may influence treatment outcome

## Discussion

Despite a consensus on increasing diversity to reduce health disparities, minimal research has examined the relationship between cultural competence, workforce diversity, and SUD treatment quality for minoritized populations. Only nine studies investigating this relationship were identified, with five published by the same author and conflicting findings [35–39]. Some studies report a positive relationship between workforce diversity and cultural competence and SUD treatment outcomes, whereas others find no association or adverse relationships. Additionally, comparisons of findings are threatened by variations in research methodologies, including measurement inconsistencies (e.g., population of focus, treatment outcomes, workforce measures). Moreover, existing literature largely focuses on SUD treatment quality for Hispanic/Latino populations. Few studies explored other minoritized populations [35, 37, 39]. Only one focused on racially minoritized people [40].

Finally, while most identified studies cited U.S. federal research support, a review of the specific grants found only one mention of workforce diversity in the purpose statements [39]. This suggests that previous studies were spin-off investigations of larger projects rather than the focus of the specific federal grants. This review identified a potential gap in federal funding dedicated to this topic and describes the state of research on this critical issue, highlighting opportunities for future studies.

### State of Evidence

The literature does not provide sufficient evidence of a relationship between cultural competence or workforce diversity and SUD treatment outcomes. Only two of the nine studies provide direct evidence of a positive relationship between concordance and treatment outcomes in minoritized patients [42, 43].

The broader healthcare system literature also reports conflicting findings. A systematic review examining patient and healthcare provider race concordance reported inconclusive evidence of a positive impact on health outcomes for minorities, citing significant variations in research as impacting interpretations [18]. Although patient satisfaction tends to be higher with racial and ethnic concordance between patients and providers, [45] it does not explain disparities in access to treatments across all contexts [46]. Differences in findings may be attributed to variations in study outcomes.

Three studies found evidence that workforce factors other than diversity may improve outcomes [35–37]. These included language translators, [36] program managers with culturally sensitive beliefs [35], and staff community involvement, measured by self-reported interaction with the community [37]. These findings suggest that other workforce characteristics may be essential in improving SUD treatment outcomes and quality in minoritized populations.

The finding that staff community involvement and availability of language translators help improve treatment outcomes, while staff diversity does not, suggests that personal commitment and culturally relevant skill of treatment staff may be more important than their race or ethnicity. Additionally, treatment program managers’ culturally responsive beliefs improved treatment outcomes, whereas organizational implementation of culturally competent practices does not; this highlights the critical role of leadership in SUD treatment. This finding is supported by broader literature on SUD treatment and the role of leadership [44]. Research is needed to clarify which workforce characteristics are most integral in SUD treatment quality for minoritized populations.

### Inconsistency in the Literature

Although all studies examined SUD treatment outcomes, there was little consistency in the definitions and measurements of said outcomes. Included studies used patient self-reports, program reports, or clinician reports. Only one study provided validity evidence, including a biomarker validating patient-self-reported substance use [43]; all other studies relied on self-reports. Although self-reported data are valid, they can be subject to social desirability reporting bias [47, 48]. Data standards have been identified as a priority by the IOM’s Committee on Data Standards and Patient Safety and are a necessary component in healthcare research to produce reliable and valid evidence, as well as to share results across facilities, providers, organizations, and other entities [49].

Additionally, no standardized measures were identified in the literature to measure cultural competency. Four of the nine articles written by the same author used the same measures [35–38]. Although the Culturally and Linguistically Appropriate Services standards (CLAS) provide health and healthcare organizations with action steps for meeting the needs of individuals from culturally and linguistically diverse backgrounds, no standardized approach to cultural competency from a practical or educational level exists [50].

Evidence also suggests that there is little consistency in the accountability measures for implementing standards or training, with few guidelines for determining the effectiveness of such interventions. Although standards for cultural competency exist across many health professions disciplines, the language used in these standards differs (i.e., culturally responsive care, cultural sensitivity) [51–54]. Therefore, cultural competence in education is ubiquitous. However, this is not the case regarding health professions’ training and licensing in the U.S. Only 10 of the 50 states, District of Columbia, and territories have provisions requiring cultural competency training as part of initial licensing or continuing education requirements (CLAS standards) [55].

Lastly, the CLAS standards are minimally represented in SUD treatment and policy [56]. The lack of standardization in defining cultural competency threatens the ability to consistently measure and quantify its impact on outcomes for minoritized populations [57]. Creating a consensus definition of cultural competency and associated measurement tools is needed to advance knowledge about the workforce and treatment.

### Equitable Representation in the Literature

The identified literature largely focused on the role of workforce diversity in SUD treatment for ethnically minoritized populations, specifically Hispanic/Latinx. Racially minoritized populations, especially those who identify as Black, were examined in less than half of the studies and only when parallel to the exploration of ethnicity. Notably, race and ethnicity are not mutually exclusive. The lack of research focusing on racial minorities is concerning, as SUD-related deaths disproportionately affect these populations.

In 2022, the Centers for Disease Control and Prevention (CDC) reported alarming statistics on drug related overdose deaths among non-Hispanic Black Americans. Between 2019 and 2020, the rate of drug overdose deaths among this population climbed from 39 to 44%. [1] More alarming is the fact that these statistics do not track the burden of SUD across the population. Non-Hispanic Black Americans are reported to have similar rates of SUD to White Non-Hispanic Americans (17.2% and 17.0%, respectively, in 2021), but their death rate is nearly seven times higher [58]. Longstanding disparities in access to treatment are well documented and are likely significant contributors to the burden [59–61]. The lack of studies exploring workforce diversity and treatment quality for Black Americans is concerning, given the rate of overdose deaths. Researchers and policymakers must prioritize research on the impact of workforce diversity on racial minorities.

The number of identified studies focusing on ethnic minorities may reflect their representation as a population group. As of 2023, the U.S. Census reports Hispanic/Latinx people constitute 18.7% of the population [62]. In 2023, Hispanic/Latinx youth accounted for over a quarter of the population under 18 years old [62]. As the population of Hispanic/Latinx Americans increases, research must investigate the role of workforce diversity and the quality of SUD treatment in this community to better inform policies.

Minoritized populations absent from this research must be noted. This review found no research examining workforce characteristics and outcomes for American Indian/Alaskan Native (AI/AN) people [1]. However, this population has the second-highest reported rate of SUD-related deaths, behind Black people [63, 64]. Notably, the rates of substance use among multiracial people are higher than those among Black or Hispanic/Latinx people [65, 66]. Multiracial people are projected to be the fastest-growing racial group over the next several decades, followed by Asians and Hispanic/Latinx people [59]. With the U.S. population becoming more diverse, public health surveillance systems must prioritize minoritized populations to further understand their preferences, needs, and treatment outcomes.

### Research Funding

The NIH established the Office of Minority Programs, now the National Institute on Minority Health and Health Disparities (NIMHD) in 2010 to advance research in health disparities, investing nearly $3.3 billion [32]. Similarly, the NIH has spent over $17 billion to advance science on drug use and addiction through the National Institute on Drug Abuse (NIDA) and the National Institute on Alcohol Abuse and Alcoholism (NIAAA). The importance of workforce diversity and minoritized populations has also been acknowledged through the formation of workgroups in the NIDA, such as the Racial Equity Initiative Scientific Workforce Diversity group [67]. Even with understanding of and commitment to improving racial equity in healthcare, the current study identified only one funded study focused on the relationship between workforce diversity and SUD treatment.

According to the Agency for Healthcare and Quality (AHRQ), lack of concordance between patient and providers could produce lower quality healthcare due to stereotyping, stigma, or miscommunications. The lack of research funding appears to be a significant oversight and could fuel discriminatory practices in care [68].

There is also a lack of funding from other sources [69]. Although AHRQ’s report acknowledges a link between patient-provider concordance and health disparities, there is no evidence that they prioritize this research [61]. Similarly, the Health Resources and Services Administration’s (HRSA) Bureau of Health Workforce (BHW) [70] and Substance Abuse and Mental Health Services Administration (SAMHSA) [71] are dedicated to equitable care for high-need communities by strengthening the health workforce; however, they demonstrate similar oversight.

### Policy Implication and Call to Action

Despite progress in healthcare delivery and education as outlined in Crossing the Quality Chasm, the alarming increase in overdose deaths among Black Americans highlights a significant lack of progress in addressing SUD treatment and associated disparities for minoritized populations [5, 72]. While this issue has not always received the focused attention it deserves from policymakers, researchers, and academic leaders, there is a growing recognition of its importance. A sustained and proactive commitment to addressing it will be essential for meaningful progress.

*Unequal Treatment* called for data collection and monitoring of health disparities, as well as research to identify the source of these disparities and promising intervention strategies [7]. To build a knowledge base on SUD, workforce characteristics, and treatment outcomes, we propose the following calls to action for policymakers, federal agencies, nongovernmental organizations, researchers, and academic leaders:

- Consensus Definition of Cultural Competence: The National Academies of Sciences, Engineering, and Medicine (NASEM) should prepare a report on cultural competence in healthcare and recommend a consensus definition. [73] Federal agencies (NIH, AHRQ, SAMHSA, CDC, etc.) and nongovernmental organizations (Institute for Health Improvement, American Public Health Association, etc.) should collaborate to create a national definition and oversee the development of associated measurement tools. [74, 75] This definition should be adopted across academic, research, and healthcare systems. [76]
- Dedicated Research Funding: Congress should appropriately fund federal research sponsors (NIH, AHRQ, SAMSHA, and HRSA) and nongovernmental organizations. [77] Philanthropic and non-profit organizations should invest in research examining characteristics of the health workforce that impact SUD treatment outcomes and quality.
- Collaborative Commitment to Advancing Equity: Researchers should prioritize studies of minoritized populations most affected by SUD, including Black and AI/AN people. [78–81] National surveillance systems, including the CDC, should enhance SUD monitoring for growing minoritized populations, including multiracial populations.
- Unified Cultural Competency Education Standards: Education accreditors, including the Academy of American Medical Colleges, American Association of Colleges of Nursing, Council on Social Work Education, American Psychological Association, and others should adopt unified standards for consistency across programs. [82, 83]
- State and territorial policymakers should adopt provisions requiring cultural competency training for licensure and continuing education in regulated healthcare occupations. [56, 84, 85]

## Supplementary Information

Additional file 1

## Data Availability

No datasets were generated or analysed during the current study.

## Abbreviations

SUD: Substance Use Disorder
U.S.: United States
IOM: Institute of Medicine
PRISMA: Preferred Reporting Items for Systematic Review and Meta-Analyses
MeSH: Medical Subject Headings
NLM: National Library of Medicine
RePORTER: Research Portfolio Online Reporting Tool
NIH: National Institute of Health
CLAS: Culturally and Linguistically Appropriate Services
CDC: Centers for Disease Prevention and Control
AI/AN: American Indian/Alaskan Native
NIMHD: National Institute on Minority Health and Health Disparities
NIDA: National Institute on Drug Abuse
NIAAA: National Institute on Alcohol Abuse and Alcoholism
AHRQ: Agency for Healthcare and Quality
HRSA: Health Resources and Services Administration
BHW: Bureau of Health Workforce
SAMHSA: Substance Abuse and Mental Health Services Administration
NASEM: National Academies of Sciences, Engineering, and Medicine
