# Supplementary material for: Substance use disorder, the workforce, and treatment quality for minoritized populations: a systematic review
Source: Subst Abuse Treat Prev Policy. 2025 Jun 19;20:26. doi: 10.1186/s13011-025-00656-8 (PMC12178033; doi:10.1186/s13011-025-00656-8)
Supplement: Supplementary file 1 — Additional file 1 [file 13011_2025_656_MOESM1_ESM.docx]

**EXHIBIT A1.** Search queries of MeSH terms

| (Quality of Health Care) AND "Substance-Related Disorders"[Mesh] |
| --- |
| ("Quality Indicators, Health Care"[Mesh]) AND "Cultural Diversity"[Mesh] |
| (("Quality of Health Care"[Mesh]) AND "Health Workforce"[Mesh]) AND "Diversity, Equity, Inclusion"[Mesh] |
| (("Quality Indicators, Health Care"[Mesh]) AND "Health Workforce"[Mesh]) AND "Diversity, Equity, Inclusion"[Mesh] |
| ("Quality Indicators, Health Care"[Mesh]) AND "Diversity, Equity, Inclusion"[Mesh] |
| (("Quality Indicators, Health Care"[Mesh]) AND "Health Workforce"[Mesh] |
| (("Quality Indicators, Health Care"[Mesh]) AND "Health Care Quality, Access, and Evaluation"[Mesh]) AND "Health Workforce"[Mesh] |
| ("Cultural Competency"[Mesh]) AND "Health Workforce"[Mesh] |
| (("Quality Assurance, Health Care"[Mesh]) AND "Substance-Related Disorders"[Mesh]) AND "Health Workforce"[Mesh] |
| (("Opioid-Related Disorders"[Mesh]) AND "Quality of Health Care"[Mesh]) AND "Cultural Diversity"[Mesh] |
| (("Racial Groups"[Mesh]) AND "Substance-Related Disorders"[Mesh]) AND "Health Workforce"[Mesh] |
| ("Cultural Competency"[Mesh]) AND "Substance-Related Disorders"[Mesh] |
| (("Cultural Diversity"[Mesh]) AND "Substance-Related Disorders"[Mesh]) AND "Treatment Outcome"[Mesh] |
| (("Quality Indicators, Health Care"[Mesh]) AND "Health Workforce"[Mesh]) AND "Cultural Diversity"[Mesh] |
| (("Quality of Health Care"[Mesh]) AND "Substance-Related Disorders"[Mesh]) AND "Health Workforce"[Mesh] |
| (("Quality Indicators, Health Care"[Mesh]) AND "Health Workforce"[Mesh]) AND "Cultural Diversity"[Mesh] |
| (("Quality Indicators, Health Care"[Mesh]) AND "Health Workforce"[Mesh]) AND "Culturally Competent Care"[Mesh] |
| (("Quality Indicators, Health Care"[Mesh]) AND "Health Workforce"[Mesh]) AND "Cultural Competency"[Mesh] |
| ((("Quality Indicators, Health Care"[Mesh]) AND "Health Workforce"[Mesh])) AND "Cultural Diversity"[Mesh] |
| ((("Quality of Health Care"[Mesh]) AND "Health Workforce"[Mesh]) AND "Substance-Related Disorders"[Mesh]) AND "Diversity, Equity, Inclusion"[Mesh] |
| (("Substance-Related Disorders"[Mesh]) AND "Health Workforce"[Mesh]) AND "Diversity, Equity, Inclusion"[Mesh] |
| (("Substance-Related Disorders"[Mesh]) AND "Health Workforce"[Mesh]) AND "Culturally Competent Care"[Mesh] |
| (("Substance-Related Disorders"[Mesh]) AND "Health Workforce"[Mesh]) AND "Cultural Competency"[Mesh] |
| (("Substance-Related Disorders"[Mesh]) AND "Health Workforce"[Mesh]) AND "Clinical Competence"[Mesh] |
| ((("Quality of Health Care"[Mesh]) AND "Health Workforce"[Mesh]) AND "Diversity, Equity, Inclusion"[Mesh]) AND "Substance-Related Disorders"[Mesh] |
| (("Health Personnel"[Mesh]) AND "Quality of Health Care"[Mesh]) AND "Substance-Related Disorders"[Mesh] |
| ((("Health Personnel"[Mesh]) AND "Quality of Health Care"[Mesh]) AND "Substance-Related Disorders"[Mesh]) AND "Diversity, Equity, Inclusion"[Mesh] |
| (("Health Personnel"[Mesh]) AND "Quality Indicators, Health Care"[Mesh]) AND "Substance-Related Disorders"[Mesh] |
| ((("Health Personnel"[Mesh]) AND "Quality Indicators, Health Care"[Mesh]) AND "Substance-Related Disorders"[Mesh]) AND "Diversity, Equity, Inclusion"[Mesh] |
| (("Health Personnel"[Mesh]) AND "Substance-Related Disorders"[Mesh]) AND "Culturally Competent Care"[Mesh] |
| (("Health Personnel"[Mesh]) AND "Substance-Related Disorders"[Mesh]) AND "Cultural Competency"[Mesh] |
| (("Health Personnel"[Mesh]) AND "Substance-Related Disorders"[Mesh]) AND "Clinical Competence"[Mesh] |
| (("Health Personnel"[Mesh]) AND "Substance-Related Disorders"[Mesh]) AND "Clinical Competence"[Mesh] |
| (("Quality Indicators, Health Care"[Mesh]) AND "Substance-Related Disorders"[Mesh]) AND "Treatment Adherence and Compliance"[Mesh] |
| ((("Quality Indicators, Health Care"[Mesh]) AND "Substance-Related Disorders"[Mesh]) AND "Health Workforce"[Mesh]) AND "Treatment Adherence and Compliance"[Mesh] |
| ((("Quality Indicators, Health Care"[Mesh]) AND "Substance-Related Disorders"[Mesh]) AND "Health Personnel"[Mesh]) AND "Treatment Adherence and Compliance"[Mesh] |
| ((("Quality of Health Care"[Mesh]) AND "Substance-Related Disorders"[Mesh]) AND "Treatment Adherence and Compliance"[Mesh]) AND "Health Workforce"[Mesh] |
| ((("Health Personnel"[Mesh]) AND "Substance-Related Disorders"[Mesh]) AND "Treatment Adherence and Compliance"[Mesh]) AND "Quality of Health Care"[Mesh] |
| ((("Quality of Health Care"[Mesh]) AND "Substance-Related Disorders"[Mesh]) AND "Healthcare Disparities"[Mesh]) AND "Health Personnel"[Mesh] |
| ((("Quality of Health Care"[Mesh]) AND "Substance-Related Disorders"[Mesh]) AND "Healthcare Disparities"[Mesh]) AND "Health Workforce"[Mesh] |
| ((("Quality of Health Care"[Mesh]) AND "Substance-Related Disorders"[Mesh]) AND "Diversity, Equity, Inclusion"[Mesh]) AND "Health Workforce"[Mesh] |
| ((("Quality of Health Care"[Mesh]) AND "Substance-Related Disorders"[Mesh]) AND "Cultural Diversity"[Mesh]) AND "Health Workforce"[Mesh] |
| ((("Quality of Health Care"[Mesh]) AND "Health Workforce"[Mesh]) AND "Treatment Outcome"[Mesh]) AND "Substance-Related Disorders"[Mesh] |
| ((("Quality of Health Care"[Mesh]) AND "Health Personnel"[Mesh]) AND "Treatment Outcome"[Mesh]) AND "Substance-Related Disorders"[Mesh |
